# Examining racial and ethnic disparities in adult emergency department patient visits for concussion in the United States

**DOI:** 10.3389/fneur.2022.988088

**Published:** 2022-09-30

**Authors:** Landon B. Lempke, Zachary Yukio Kerr, Patrice Melvin, Samuel R. Walton, Jessica S. Wallace, Rebekah C. Mannix, William P. Meehan, Valerie L. Ward

**Affiliations:** ^1^Division of Sports Medicine, Boston Children's Hospital, Boston, MA, United States; ^2^The Micheli Center for Sports Injury Prevention, Waltham, MA, United States; ^3^Michigan Concussion Center, School of Kinesiology, University of Michigan, Ann Arbor, MI, United States; ^4^Department of Exercise and Sport Science, The University of North Carolina at Chapel Hill, Chapel Hill, NC, United States; ^5^Office of Health Equity and Inclusion, The Sandra L. Fenwick Institute for Pediatric Health Equity and Inclusion, Boston Children's Hospital, Boston, MA, United States; ^6^Department of Health Science, University of Alabama, Tuscaloosa, AL, United States; ^7^Department of Emergency Medicine and Pediatrics, Harvard Medical School, Boston, MA, United States; ^8^Division of Emergency Medicine, Boston Children's Hospital, Boston, MA, United States; ^9^Office of Health Equity and Inclusion, Boston Children's Hospital, Boston, MA, United States; ^10^Sandra L. Fenwick Institute for Pediatric Health Equity and Inclusion, Boston Children's Hospital, Boston, MA, United States; ^11^Department of Radiology, Boston Children's Hospital, Boston, MA, United States; ^12^Harvard Medical School, Boston, MA, United States

**Keywords:** gender, healthcare, socioeconomic status, health equity, epidemiology, mild traumatic brain injury

## Abstract

**Background:**

Racial and ethnic differences in emergency department (ED) visits have been reported among adolescent patients but are unsubstantiated among adults. Therefore, our purpose in this study was to examine the relationship between race/ethnicity and adult ED visits for concussions, their injury mechanisms, and computed tomography (CT) scan use among a nationally representative sample.

**Methods:**

We used the National Hospital Ambulatory Medical Care Survey database from 2010–2015 to examine 63,725 adult (20–45 years old) patient visits, representing an estimated 310.6 million visits presented to EDs. Of these visits, 884 (4.5 million national estimate) were diagnosed with a concussion. Visit records detailed patient information (age, sex, race/ethnicity, geographic region, primary payment type), ED visit diagnoses, injury mechanism (sport, motor vehicle, fall, struck by or against, “other”), and head CT scan use. The primary independent variable was race/ethnicity (non-Hispanic Asian, non-Hispanic Black or African American, Hispanic/Latinx, non-Hispanic multiracial or another, and non-Hispanic White). We used multivariable logistic and multinomial regression models with complex survey sampling design weighting to examine the relationship between concussion ED visits, injury mechanisms, and CT scan use separately by race/ethnicity while accounting for covariates.

**Results:**

There were no associations between race/ethnicity and concussion diagnosis among adult ED visits after accounting for covariates. Relative to sports-related injuries, non-Hispanic Black or African American patient visits were associated with a motor vehicle (OR = 2.69, 95% CI: 1.06–6.86) and “other” injury mechanism (OR = 4.58, 95% CI: 1.34–15.69) compared to non-Hispanic White patients. Relative to sports-related injuries, non-Hispanic Asian, multiracial, or patients of another race had decreased odds of falls (OR = 0.20, 95% CI: 0.04–0.91) and “other” injuries (OR = 0.09, 95% CI: 0.01–0.55) compared to non-Hispanic White patients. The odds of a CT scan being performed were significantly lower among Hispanic/Latinx patient visits relative to non-Hispanic White patients (OR = 0.52, 95% CI: 0.30–0.91), while no other race/ethnicity comparisons differed.

**Conclusion:**

Our findings indicate that the overarching concussion ED visit likelihood may not differ by race/ethnicity in adults, but the underlying mechanism causing the concussion and receiving a CT scan demonstrates considerable differences. Prospective future research is warranted to comprehensively understand and intervene in the complex, multi-level race/ethnicity relationships related to concussion health care to ensure equitable patient treatment.

## Introduction

Traumatic brain injuries are common, with over 223,000 hospitalizations in the United States in 2019 (1). Most traumatic brain injuries are concussions and with 32% of concussion patients seeking outpatient health care (2) and accounting for 2.5 million emergency department (ED) visits annually (3). Given the concussion healthcare burden, identifying areas of disparity in the ED is essential for identifying, creating, and implementing interventions to ensure equitable healthcare provision (4). This is especially important when considering the complex inequities in health care access and delivery, especially as it relates to the ED. For example, relative to non-Hispanic White patients, non-Hispanic Black or African American and Hispanic/Latinx patients more frequently use the ED as their primary health care source (5), have lower trust in health care professionals (6), and experience 6–18% longer ED visit wait times (7). Thus, examining potential disparities surrounding concussion ED visits is necessary to achieve more equitable health care access and delivery.

Concussion-related racial/ethnic disparities in ED visits have been examined in three ways. First, prior work among adolescents has demonstrated that non-Hispanic Black or African American patients had 3–4 times the odds of a concussion being attributed to assault relative to the sport than non-Hispanic White patients (8). Although the mechanism of injury for concussion ED visits among adults has been examined previously, (3) such findings have not been stratified by race/ethnicity. Second, limited findings relate to racial/ethnic disparities associated with presenting to an ED. Findings pertaining to adolescent ED visits *via* ambulatory services have observed non-Hispanic Black or African American patients having lower odds of concussion ED visits than non-Hispanic White patients (8, 9). Third, the decision to receive a head computed tomography (CT) scan has also been associated with race/ethnicity, such that Hispanic/Latinx and non-Hispanic Black or African American adolescent patients have lower odds of receiving a CT scan than non-Hispanic White patients (10). Although a head CT scan cannot be used to diagnose a concussion (11), it can be used to rule out structural neurotraumatic injuries and the medical decision to receive one among individuals with a concussion brings financial and radiation exposure concerns that may impact individuals of different racial/ethnic backgrounds. Though the cumulative work (5–10) suggests racial/ethnic inequity nationally and among adolescents seeking care for a concussion at the ED, examining concussion ED visits among adults is warranted to better understand if these same patterns persist as individuals transition from adolescence to adulthood.

Adulthood confers increased autonomy over life choices, such as health care-seeking behaviors. Specifically, due to medical autonomy, adults can make their own decision to either receive or refuse ambulatory healthcare services after a suspected concussion. In contrast, adolescents are often bound by parental decisions or even the legislature concerning concussions (12). Further, compared to adolescents, sports are a less common reason for adult ED visits (2, 3, 13, 14). Other activities such as resuming driving, work, or general activities of daily living are considered less with inconsistent guidance (15). These activities may leave patients more vulnerable to secondary injury (16) or longer recovery if a sub-symptom activity is not followed (17). Thus, only examining and understanding concussion ED visits among children and adolescents (8) may not translate to adult patients, leaving race/ethnicity-based patterns of concussion in adult patients undetermined.

The Health Disparities Framework provides strong guidance for research progression by (1) detecting, (2) understanding, and (3) reducing the problem through interventional approaches (4). Determining if the same disparities are present in adulthood as in childhood (8) for concussion health care is an essential first step in identifying whether a problem exists. Specifically, we must establish if racial/ethnic differences exist in adult concussion ED visits, the associated mechanism of injury, or the medical evaluation (e.g., CT scan use) to understand the problem better. Elucidating the underlying concussion ED visit problem can, in turn, lead to targeted intervention strategies, such as focused education (18–20) and resource allocation (21), to resolve the inequity. Thus, establishing if racial/ethnic differences exist among adult concussion ED visits is necessary to tailor healthcare toward specific groups to improve patient health outcomes ultimately.

Using data from the National Hospital Ambulatory Medical Care Survey (NHAMCS) database between 2010 and 2015, our study aimed to determine if racial/ethnic disparities existed within ED concussion visits among adults aged 20–45. We focused on differences related to (1) the reported number of concussion visits, (2) the distribution of concussion injury mechanisms, and (3) the use of a head CT scan as a service completed during the concussion ED visit. Our study is based on a similar methodology as Wallace and Mannix (8), who examined *children and adolescents* (0–19 years old) using the same database and years. Based on this prior work and others (8, 10), we hypothesized that those not identified as non-Hispanic White would be less likely to have ED visits for concussion, more likely to sustain a concussion from being struck or sustaining a fall compared to a sport, and less likely to receive a CT scan compared to non-Hispanic White patients.

## Methods

This study used a retrospective, cross-sectional design across 6 years (2010–2015) from the National Hospital Ambulatory Medical Care Survey (NHAMCS) database (22). The NHAMCS is an annual, national probability sample of ambulatory visits made to non-federal general and short-stay hospitals in the United States. It has been conducted by the National Center for Health Statistics (NCHS) since 1992. NHAMCS data were collected from a three-stage ED sample design across the entire United States consisting of (1) 112 geographic primary sampling units that comprise a probability subsample of primary sampling units from the initial 1985 to 1994 National Health Interview Surveys (23); (2) 449–640 hospitals within primary sampling units (number varies by survey year); and (3) patient visits within all of the emergency service areas within sampled EDs. Hospitals included in the sample were randomly assigned to 16 panels with 4-week reporting periods, resulting in each included hospital being surveyed approximately once every 15 months. Further details about NHAMCS data collection methods for each survey year can be found in established documentation reported by NCHS (24).

The NHAMCS database included ED visits from an annual, standardized survey instrument used to collect patient use and provision of ambulatory care services in hospital EDs. The NHAMCS data collection was carried out by hospital staff and Census Bureau field representatives who extracted data from medical records using a paper-based questionnaire in 2010 and 2011 and computerized questionnaires from 2012 to 2015. Data obtained on patients include characteristics such as age, sex, race, ethnicity, the reason for the visit, the provider's diagnosis, services ordered or provided (e.g., CT scan), and treatment. NHAMCS is approved annually by the NCHS Ethics Review Board with waivers of the requirements to obtain the informed consent of patients and patient authorization for the release of patient medical record data by health care providers, and institutional review board approval is not required for deidentified secondary data analysis of publicly available surveillance data.

### Concussion, injury mechanism, and CT scan patient record identification

Population-weighted patient visits to the ED among young to middle-aged adults aged 20–45 years were included in the analysis. Adults >45 years old were excluded due to known increased concussion odds among older adults (2, 3). The age inclusion parameter produced a cohort of 63,725 adult patient ED visits, representing an estimated 310,621,000 adult ED visits nationally within the last 6 years. Participants were categorized into those with and without a concussion diagnosis based on standard International Classification of Diseases (ICD-9-CM) codes. Concussion diagnosis was treated as a binary (yes or no) variable based on at least one of the following ICD-9-CM code parameters being entered into 1 of 3 potential diagnosis codes using the code range of 850.0–850.12 and the specific code 959.01. Importantly, 959.01 (head injury not specified) was included to encapsulate misidentified concussions and provide identical methods to Wallace and Mannix (8). The concussion injury mechanism was identified using the designated ICD-9-CM external cause of injury codes (E-codes) for each patient (8). We categorized the injury mechanisms into five categories: (8) sports (E001-E010), falls (E880-E888), motor vehicle collisions (E810-E825), being struck by or against (E916-E917, E960.0, E968.2, E973, E975), and other (those not containing the aforementioned E-codes). Head CT scan use was treated as binary (yes or no) based on whether the medical records indicated the imaging procedure was performed.

### Race/ethnicity identification

Race and ethnicity were reported separately through the NHAMCS survey and then imputed and combined into a single variable by the NHAMCS data management team due to some data missingness. Race/ethnicity was categorized as non-Hispanic White, non-Hispanic Asian, non-Hispanic Black or African American, Hispanic/Latinx, and non-Hispanic Multiracial or another race (individuals of multiple races or those not falling into one of the other categories) (8). Non-Hispanic Whites were treated as the referent group for all analyses because they comprise the majority of ED patient visits.

### Covariate identification

We included sex, primary payment type, and geographic location due to their known associations with the outcomes and to replicate work done among children and adolescents by Wallace and Mannix (8). Sex was treated as a binary variable (female or male). The primary payment method served as a potential socioeconomic status proxy due to the differing financial constraints of each payment method. Primary payment types were split into four categories: Medicare/Medicaid, private insurance, self-pay, and others. Each ED visit's geographic region was recorded and categorized through the NHAMCS dataset as Northeast, Midwest, South, or West based on United States Census Bureau reports. The geographic region was not included as a statistical modeling covariate but was used for descriptive purposes.

### Statistical analysis

Survey data were analyzed using the sampled visit weight *via* the inverse of the selection probabilities. NCHS adjusted the sampling weights for survey non-response within the time of year, geographic region, urban/rural, and ownership designations, yielding an unbiased national estimate of ED visit occurrences, percentages, and characteristics.

Descriptive statistics consisting of sample frequency, population-weighted national estimates (rounded to the nearest 1,000 per standard NCHS recommendations), and population-weighted proportions with 95% confidence intervals (95% CI) for all variables were calculated by whether a concussion diagnosis occurred. Descriptive statistics specific to those only with a concussion diagnosis were cross-tabulated by race/ethnicity, sex, geographic region, primary payment type, injury mechanism, and CT scan use. The associations between race/ethnicity and concussion diagnosis (yes vs. no) and CT scan use (yes vs. no) were analyzed separately using univariable and multivariable logistic regression models while accounting for sex and primary payment type (8). A multivariable, multinomial logistic regression model was used to examine the association between race/ethnicity and injury mechanism only among adults with a concussion diagnosis, accounting for sex and primary payment type. The non-Hispanic Asian and non-Hispanic multiracial or other race categories had limited observations to draw accurate population estimates or complete planned statistical analyses. They were, therefore, combined when describing and examining injury mechanism associations into a “non-Hispanic Asian, multiracial, or other” category. Odds ratios (OR) with 95% CI were derived from the logistic regression models, with 95% CI values not including 1.0 considered statistically significant. All analyses were performed using SAS version 9.4 (SAS Institute Inc., Cary, NC, USA) using established complex survey sampling design packages.

## Results

We observed 884 adult ED visits for concussions from the 62,841 total ED visits, representing a total population visit estimate of *N* = 4,505,000 concussions across the 2010–2015 timeframe (Table 1). The highest proportion of concussion ED visits across included variables independently occurred among men (*N* = 2,605,000; 57.8%), non-Hispanic White patients (*N* = 2,717,000; 60.3%), in the South geographic location (*N* = 1,394,000; 30.9%), and primary payment type being private insurance (*N* = 1,606,000; 35.7%; Table 1).

**Table 1:** Descriptive characteristics of adult emergency department visits by concussion and no-concussion diagnosis (*n* = 63,725; est = 310,621,000).

	**Concussion diagnosis**				**No concussion diagnosis**			
	**(*****n*** = **884; Population estimate** = **4,505,000)**				**(*****n*** = **62,841; Population estimate** = **306,117,000)**			
**Demographic characteristics**	**Frequency**	**Population estimate**	**Population proportion (%)**	**(Population 95% CI)**	**Frequency**	**Population estimate**	**Population proportion (%)**	**(Population 95% CI)**
**Sex**								
Female	379	1,900,000	42.2	(37.5, 46.9)	37,143	181,994,000	59.5	(58.7, 60.2)
Male	505	2,605,000	57.8	(53.1, 62.5)	25,698	124,122,000	40.5	(39.8, 41.3)
**Race/ethnicity**								
Non-Hispanic Asian	21	71,000	1.6	(0.7, 2.5)	1,062	3,998,000	1.3	(1.1, 1.5)
Non-Hispanic Black or African American	153	855,000	19.0	(14.6, 23.4)	15,159	75,815,000	24.8	(22.4, 27.1)
Hispanic/latinx	158	766,000	17.0	(13.2, 20.8)	9,829	46,116,000	15.1	(13.6, 16.5)
Non-Hispanic multiracial or another	17	95,000	2.1	(0.9, 3.4)	1,060	4,758,000	1.6	(1.2, 1.9)
Non-Hispanic white	535	2,717,000	60.3	(55.1, 65.6)	35,731	175,429,000	57.3	(55.1, 59.5)
**Geographic region**								
Northeast	220	957,000	21.2	(16.9, 25.6)	13,383	52,546,000	17.2	(14.2, 20.1)
Midwest	193	985,000	21.9	(16.7, 27.0)	14,759	72,557,000	23.7	(19.8, 27.6)
South	256	1,394,000	30.9	(25.6, 36.3)	22,088	118,405,000	38.7	(34.3, 43.1)
West	215	1,168,000	25.9	(20.7, 31.2)	12,611	62,609,000	20.5	(17.3, 23.6)
**Primary payment type**								
Private insurance	335	1,606,000	35.7	(30.3, 41.0)	19,152	93,481,000	30.5	(29.4, 31.7)
Medicare/medicaid	199	946,000	21.0	(16.8, 25.2)	21,260	99,471,000	32.5	(31.2, 33.8)
Self-pay	200	1,066,000	23.7	(18.6, 28.8)	13,044	66,357,000	21.7	(20.4, 22.9)
Work's comp./ no charge/ other	90	496,000	11.0	(8.0, 14.0)	3,945	18,874,000	6.2	(5.4, 6.9)
Blank/unknown^a^	60	391,000	8.7	(5.3, 12.1)	5,440	27,933,000	9.1	(7.8, 10.5)
**Injury-related**								
Yes	884	4,505,000	100.0	(100, 100)	17,542	84,726,000	27.7	(27.0, 28.3)
**Injury mechanism^b^**								
Sports related	40	221,000	4.9	(3.0, 6.9)	864	4,034,000	4.8	(4.2, 5.3)
Falls	192	996,000	22.1	(17.8, 26.4)	2,678	13,113,000	15.5	(14.6, 16.3)
Motor vehicle	238	1,225,000	27.2	(22.2, 32.1)	2,346	11,904,000	14.0	(13.3, 14.8)
Struck by or against^c^	276	1,264,000	28.1	(23.9, 32.2)	1,883	8,891,000	10.5	(9.8, 11.1)
Other	138	799,000	17.7	(13.4, 22.1)	9,771	46,786,000	55.2	(54.2, 56.3)
**CT Scan use**								
Yes	648	3,294,000	73.1	(68.6, 77.6)	8,127	41,069,000	13.4	(12.8, 14.0)

a Blank/unknown (n = 5,550) pay status was excluded from subsequent modeling.

b Among injury-related ED visits.

c Struck by or against injuries include: Struck accidentally by a falling object; striking against or struck accidentally by a person or objects; injury due to legal intervention by the blunt object or by other specified means; unarmed fight or brawl; assault by striking by a blunt or thrown object.

### Adult concussion ED visit descriptive statistics

Adult concussion ED visit proportions tabulated across race/ethnicity by sex were relatively consistent, with men making up the majority of visits (56.7–67.3%; Table 2). The majority of concussion visits took place in the South geographic region among non-Hispanic Black or African American (*N* = 357,000; 41.8%) and non-Hispanic White patients (*N* = 835,000; 30.7%), and in the West geographic location for Hispanic/Latinx (*N* = 380,000; 49.7%) and non-Hispanic Asian, multiracial, or other race patients (*N* = 81,000; 48.5%). Self-pay was the predominant concussion ED visit payment source for non-Hispanic Black or African American (*N* = 289,000; 33.8%) and Hispanic patients (*N* = 239,000; 31.1%; Table 2), whereas private insurance was the most common for non-Hispanic White (*N* = 1,189,000; 43.7%) and non-Hispanic Asian, Multiracial, or other race patients (*N* = 61,000; 36.6%; Table 2).

**Table 2:** Adult emergency department visits with concussion across race/ethnicity (*n* = 884).

	**Non-Hispanic Black or African American**			**Hispanic/Latinx**			**Non-Hispanic White**			**Non-Hispanic Asian, multiracial, or another^a^**		
	**(*****n*** = **153; Population** **estimate** = **855,000)**			**(*****n*** = **158; Population** **estimate** = **766,000)**			**(*****n*** = **535; Population** **estimate** = **2,717,000)**			**(*****n*** = **38; Population** **estimate** = **166,000)**		
**Demographic characteristics**	**Frequency**	**Population estimate**	**Population proportion (%)**	**Frequency**	**Population estimate**	**Population proportion (%)**	**Frequency**	**Population estimate**	**Population proportion (%)**	**Frequency**	**Population estimate**	**Population proportion (%)**
**Sex**												
Female	70	350,000	40.9	61	319,000	41.6	234	1,177,000	43.3	14	54,000	32.7
Male	83	505,000	59.1	97	447,000	58.4	301	1,541,000	56.7	24	112,000	67.3
**Geographic region**												
Northeast	37	165,000	19.2	41	150,000	19.6	135	613,000	22.6	7	29,000	17.3
Midwest	44	287,000	33.6	18	73,000	9.5	129	608,000	22.4	2	17,000	10.5
South	58	357,000	41.8	34	162,000	21.2	157	835,000	30.7	7	39,000	23.7
West	14	46,000	5.4	65	380,000	49.7	114	661,000	24.3	22	81,000	48.5
**Primary payment type**												
Private insurance	40	205,000	24.0	46	152,000	19.8	234	1,189,000	43.7	15	61,000	36.6
Medicare/medicaid	49	198,000	23.2	39	234,000	30.5	104	481,000	17.7	7	32,000	19.4
Self-pay	42	289,000	33.8	46	239,000	31.1	106	513,000	18.9	6	25,000	15.2
Work's comp./ no charge/ other	15	108,000	12.6	15	68,000	8.9	54	289,000	10.6	6	31,000	18.5
Blank/unknown^b^	7	55,000	6.5	12	73,000	9.6	37	245,000	9.0	4	17,000	10.3
**Injury mechanism**												
Sports related	6	13,000	1.5	6	42,000	5.5	24	141,000	5.2	4	24,000	14.7
Fall	32	171,000	20.0	30	192,000	25.1	123	611,000	22.5	7	22,000	13.3
Motor vehicle	41	196,000	22.9	48	217,000	28.3	141	772,000	28.4	8	40,000	24.3
Struck by or against^c^	47	240,000	28.1	43	183,000	23.9	169	768,000	28.3	17	73,000	43.8
Other	27	235,000	27.5	31	132,000	17.2	78	425,000	15.6	2	7,000	3.9
**CT scan use**												
Yes	116	623,000	13.8	102	470,000	10.4	402	2,076,000	46.1	28	126,000	2.8

a Asian and multiracial or another were combined due to insufficient cases across injury mechanisms and CT scan use.

b Blank/unknown (n = 60) pay statuses were excluded from subsequent modeling.

c Struck by or against Injuries include: Struck accidentally by a falling object; striking against or struck accidentally by a person or objects; injury due to legal intervention by the blunt object or by other specified means; unarmed fight or brawl; assault by striking by a blunt or thrown object.

OR, Odds ratio; 95% CI, 95 percent confidence interval for OR.

### Associations between adult concussion ED visits and race/ethnicity

The univariable logistic regression model demonstrated an association between race/ethnicity and concussion ED visits such that non-Hispanic Black or African American patients had 27% lower odds (OR = 0.73, 95% CI: 0.55–0.96) for a concussion ED visit than non-Hispanic White patients (Table 3). No additional statistically significant odds ratios were found for comparing non-Hispanic White patients to other racial/ethnic groups. In addition, the multivariable logistic regression model examining race/ethnicity with concussion ED visits accounting for sex (*p* < 0.001) and primary payment type (*p* < 0.001), however, demonstrated no significant differences in the concussion ED visit odds among any race/ethnicity relative to non-Hispanic White patients (Table 3; *p* ≥ 0.262).

**Table 3:** Association between race/ethnicity and concussion emergency department visits.

**Race/ethnicity**	**Univariable OR**	**(95% CI)**	**Multivariable OR^a^**	**(95% CI)**
Non-Hispanic Asian	1.15	(0.65, 2.03)	1.13	(0.64, 1.99)
Non-Hispanic Black or African American	**0.73**	**(0.55, 0.96)**	0.78	(0.60, 1.03)
Hispanic/Latinx	1.07	(0.80, 1.43)	1.09	(0.80, 1.43)
Non-Hispanic Multiracial or Another	1.29	(0.70, 2.38)	1.30	(0.70, 2.38)
Non-Hispanic White	Referent	-	Referent	-

a Multivariable ORs adjusted for sex (p < 0.001) and primary payment type (p < 0.001). Bold values indicate a statistically significant difference (p ≤ 0.05).

OR, Odds ratio; 95% CI, 95 percent confidence interval for OR.

### Concussion ED visit injury mechanism and race/ethnicity associations

The overarching concussion ED visit injury mechanism proportions differed by race/ethnicity such that being struck by or against was the highest reported for non-Hispanic Black or African American (*N* = 240,000; 28.1%) and non-Hispanic Asian, Multiracial, or other race patients (*N* = 73,000; 43.8%), whereas motor vehicles were the most common mechanism among Hispanic/Latinx (*N* = 217,000; 28.3%) and non-Hispanic White patients (*N* = 772,000; 28.4%; Table 2). The association between concussion injury mechanism and race/ethnicity demonstrated differing odds after adjusting for sex (*p* = 0.968) and primary payment type (*p* = 0.003; Table 4). Relative to non-Hispanic White patients, non-Hispanic Black or African American patients had significantly higher odds of sustaining a concussion from a motor vehicle (OR = 2.69, 95% CI: 1.06–6.86) and other injury types (OR = 4.58, 95% CI: 1.34–15.69) compared to sport. Additionally, non-Hispanic Asian, multiracial, or other race patients had significantly lower odds of sustaining a concussion from a fall (OR = 0.20, 95% CI: 0.04–0.91) or another injury type (OR = 0.09, 95% CI: 0.01–0.55) compared to sport (Table 4). No other significant associations in injury mechanisms by race/ethnicity were observed.

**Table 4:** Associations between race/ethnicity and injury mechanism for adult concussion emergency department visits.

**Race/ethnicity**	**Injury mechanism (Sport** = **Base outcome)**							
	**Motor vehicle**		**Fall**		**Struck by or against^a^**		**Other injury**	
	**OR^b^**	**(95% CI)**	**OR^b^**	**(95% CI)**	**OR^b^**	**(95% CI)**	**OR^b^**	**(95% CI)**
Non-Hispanic Asian, Multiracial, or Another^c^	0.30	(0.07, 1.31)	**0.20**	**(0.04, 0.91)**	0.52	(0.14, 1.94)	**0.09**	**(0.01, 0.55)**
Non-Hispanic Black or African American	**2.69**	**(1.06, 6.86)**	2.41	(0.72, 8.11)	2.62	(0.88, 7.84)	**4.58**	**(1.34, 15.69)**
Hispanic/Latinx	0.98	(0.29, 3.36)	0.82	(0.20, 3.33)	0.62	(0.19, 2.01)	0.78	(0.21, 2.91)
Non-Hispanic White	Referent	-	Referent	-	Referent	-	Referent	-

a Struck by or against Injuries include: Struck accidentally by a falling object; striking against or struck accidentally by a person or objects; injury due to legal intervention by the blunt object or by other specified means; unarmed fight or brawl; assault by striking by a blunt or thrown object.

b Multivariable ORs adjusted for sex (p = 0.968) and primary payment type (p = 0.003).

c Asian and Multiracial or another were combined due to insufficient cases across injury mechanisms. Bold values indicate a statistically significant difference (p ≤ 0.05).

OR, Odds ratio; 95% CI, 95 percent confidence interval for OR.

### Concussion ED visit CT scan use and race/ethnicity associations

A population-estimated 73.1% of ED visits diagnosed with concussion also had a head CT scan performed (Table 1), with a population estimated proportion varying from 2.8 to 46.1% across race/ethnicity categories (Table 2). Univariably, examining a CT scan completed across race/ethnicity demonstrated that Hispanic/Latinx patients were significantly less likely to receive a CT scan than non-Hispanic White patients (OR = 0.49, 95% CI: 0.27–0.88; Table 5). After accounting for sex (*p* = 0.353) and payment type (*p* = 0.393), Hispanic/Latinx patients remained as having significantly lower odds of receiving a head CT scan relative to non-Hispanic White patients (OR = 0.52, 95% CI: 0.30–0.91; Table 5).

**Table 5:** Association between race/ethnicity and CT scan use during adult concussion emergency department visits.

**Race/ethnicity**	**Univariable OR**	**(95% CI)**	**Multivariable OR^a^**	**(95% CI)**
Non-Hispanic Asian, Multiracial, or Another^b^	0.98	(0.37, 2.60)	0.99	(0.53, 1.25)
Non-Hispanic Black or African American	0.83	(0.39, 1.75)	0.86	(0.44, 1.70)
Hispanic/Latinx	**0.49**	**(0.27, 0.88)**	**0.52**	**(0.30, 0.91)**
Non-Hispanic White	Referent	-	Referent	-

a Multivariable ORs adjusted for sex (p = 0.353) and primary payment type (p = 0.393).

b Asian and Multiracial or another were combined due to insufficient cases across CT scan use. Bold values indicate a statistically significant difference (p ≤ 0.05).

OR, Odds ratio; 95% CI, 95 percent confidence interval for OR.

## Discussion

We observed an estimated 4.5 million ED visits leading to concussion diagnoses among 20–45-year-old adults over 6 years, representing 1.5% of all ED visits during that timeframe. Proportions of visits, however, varied by race/ethnicity, injury mechanism, and CT scan use and indicated patterned differences and potential disparities in seeking healthcare for a concussion at the ED (Tables 1, 2). Our findings suggest that the likelihood of concussion ED visits did not differ by race/ethnicity after accounting for sociodemographic variables of sex and primary payment method (i.e., a crude measure of socioeconomic status). However, some significant associations were observed in concussion injury mechanisms and CT scan services performed by race/ethnicity. Thus, our cumulative findings may indicate that although the frequency of concussion ED visits may not significantly differ by race/ethnicity, there are differences by race/ethnicity in the underlying mechanism causing the concussion and the medical decision for receiving a CT scan. In relation to the Health Disparities Framework, (4) our present work provides insights into the detection and understanding phases of racial and ethnic disparities for concussion-related adult ED visits on a national level. Future work, therefore, should continue to understand the problem by examining if these concussion inequities are still present at the patient- and provider-level so that future interventions can be fine-tuned and targeted.

Our unadjusted results highlighted significant associations when analyzing race/ethnicity independently for concussion ED visits, with non-Hispanic Black or African American patients having 27% lower odds of having an ED visit result in a concussion diagnosis relative to non-Hispanic White patients (Table 3). However, adjusting for sex and primary payment method demonstrated that this association no longer remained statistically significant. Prior work among adult ED concussion visits has observed varying concussion proportions based on sex and primary payment method, (3) but were only examined univariably limiting insights by race/ethnicity. In prior work on pediatric patients, Wallace and Mannix reported 34% lower odds of concussion ED visits among non-Hispanic Black patients than non-Hispanic White patients (8, 9). Their study methods also adjusted for sex and primary payment type when examining these associations, but after completing parallel associations in adults vs. children and adolescents, it appears that similar racial disparities are not observed in adult-aged patients. Rather, our results indicate that being male vs. female and the primary payment method likely contribute to the associations initially observed in adults. Direct causal or temporal understanding of our findings relative to pediatrics is not possible within the scope of this study, but future causal examinations of concussions throughout the lifespan are warranted to ensure comprehensive understanding.

Though we did not observe differing odds of overarching concussion ED visits by race/ethnicity, we did observe some differences in the underlying injury mechanism for concussions. Relative to non-Hispanic White patients, non-Hispanic Black or African American patients had 2.69 and 4.58 times the odds of motor vehicle and “other” injury mechanisms, respectively (Table 4). Further, non-Hispanic Asian, multiracial, or other race patients had reduced odds of falls and “other” injury mechanisms relative to non-Hispanic White patients. Prior work across adult ED visits has shown that traumatic brain injury mechanisms vary by demographic factors (14). Men have approximately two times the rate of sport- and recreation-related injuries than women, and pediatrics accounts for nearly 70% of all cases. However, these results were limited because race/ethnicity was not examined or considered in statistical models. Wallace and Mannix (8) observed disparities in concussion injury mechanisms among pediatric ED visits, with non-Hispanic Black patients having 3.8 times higher odds of concussions contributing to an assault mechanism vs. sport. Our findings contrast with their work (8), where we (1) observed different likelihoods for adult non-Hispanic Black or African American patients' injury mechanisms and (2) also observed lower odds among non-Hispanic Asian, multiracial, or other races (i.e., Wallace and Mannix's “other” group). Future prospective work is necessary to understand *why* these disparities were observed. Understanding the injury mechanism can allow for educational and interventional changes to reduce concussion risk and raise awareness for patients and healthcare providers to ensure accurate diagnosis occurs, as many in the ED are missed (25, 26), with a higher proportion missed when ED visits are not sport-related (27).

Racial and ethnic differences were also observed regarding a head CT scan performed among adult concussion ED and have financial, time, and radiation exposure concerns for patients. Relative to non-Hispanic White patients, Hispanic/Latinx patients were 48% less likely to receive a CT scan after adjusting for covariates. Notably, no other race/ethnicity category differed (Table 5). Our findings are similar to work among adolescent ED visits, which combined Hispanic/Latinx and non-Hispanic Black or African American patient groups and observed lower odds of receiving a CT scan than non-Hispanic White patients (10). Our study was not designed to elucidate the underlying factors contributing to this finding. However, our work highlights the need for deeper exploration and assurances in practice surrounding more equitable healthcare practices, such as access to translators to ensure proper medical communication. It is important to note that CT scans are an essential imaging technique for confirming or ruling out more severe neurotraumatic injuries, while concussions cannot be visualized (11). The considerably lower odds of Hispanic/Latinx patients may potentially avoid unnecessary administration of a CT scan for a concussion, but consequently and theoretically could result in greater likelihoods for missed diagnoses of severe neurotrauma and their negative sequelae that warrant future exploration among adults.

Our study had noteworthy limitations. Though robust and estimated visits weighted to be unbiased in sampling, (24) the findings come with the caveat that race/ethnicity was a combined variable and do not allow for deeper details and may have assumed only one race/ethnicity is present among patient visits. Further, 49–89% of concussions are estimated to be missed and not diagnosed in the ED due to their clinical diagnostic nature (25–27). This may indicate that our findings do not represent all ED concussion visits. However, we did include the ICD-9-CM code of 959.01 (head injury not specified) to potentially account for concussions not explicitly diagnosed (8).

## Conclusion

Health care inequities across race/ethnicity are widespread in healthcare, with prior reports indicating inequities surrounding adolescent concussion ED visits and the related mechanisms (8). Our findings expand upon prior work and indicate that the odds of ED visits among adults did not differ based on race/ethnicity when accounting for sex and the primary ED visit payment type. However, the underlying concussion ED visit injury mechanisms and whether a head CT scan was performed differed by race/ethnicity. Relative to non-Hispanic White patients, non-Hispanic Asian, multiracial, or other race/ethnicity had lower odds of fall-related and “other” concussion injury mechanisms than sport-related, while non-Hispanic Black or African American patients had higher odds of motor vehicle-related and “other” concussion injury mechanisms than sport-related. Head CT scan odds were considerably lower for Hispanic/Latinx patients than for non-Hispanic White patients. Our findings cumulatively may indicate that the initial decision to seek concussion healthcare in the ED may be more attributed to sex or the primary medical payment than race/ethnicity, but the mechanisms by which the concussion occurs and the subsequent medical imaging decision-making demonstrate potential racial/ethnic health care inequities warranting deeper examination. Future work should continue examining the complex biopsychosocial relationships surrounding concussions to ensure equitable health care access and delivery.

## Data availability statement

Publicly available datasets were analyzed in this study. This data can be found at: Centers for Disease Control and Prevention (CDC), National Center for Health Statistics (NCHS), National Hospital Ambulatory Medical Care Survey (NHAMCS), https://www.cdc.gov/nchs/ahcd/datasets_documentation_related.htm.

## Ethics statement

NHAMCS is approved annually by the NCHS Ethics Review Board with waivers of the requirements to obtain informed consent of patients and patient authorization for release of patient medical record data by health care providers, and institutional review board approval was not required for deidentified secondary data analysis of publicly available surveillance data. Written informed consent for participation was not required for this study in accordance with the national legislation and the institutional requirements.

## Author contributions

LL and WM contributed to conception and research question design of the study. LL and PM performed the statistical analysis. LL wrote the first draft of the manuscript. All authors contributed to manuscript revision, read, and approved the submitted version.

## Conflict of interest

Author ZK reports current and previous funding from CDC, DoD, NFL, and NIH. Author RM acknowledges support from the National Institute of Neurologic Disorders and Stroke (U01NS096835), the Eunice Kennedy Shriver National Institute of Child Health and Human Development (NS115942-01A1), the Department of Defense (W81XWH1920011), and Abbott Pharmaceuticals. Author WM receives royalties from (1) ABC-Clio publishing for the sale of his books, Kids, Sports, and Concussion: A guide for coaches and parents and Concussions; (2) Springer International for the book Head and Neck Injuries in Young Athletes and (3) Wolters Kluwer for working as an author for UpToDate separate from this study. His research was funded, in part, by philanthropic support from the National Hockey League Alumni Association through the Corey C. Griffin Pro-Am Tournament and a grant from the National Football League. The remaining authors declare that the research was conducted in the absence of any commercial or financial relationships that could be construed as a potential conflict of interest.

## Publisher's note

All claims expressed in this article are solely those of the authors and do not necessarily represent those of their affiliated organizations, or those of the publisher, the editors and the reviewers. Any product that may be evaluated in this article, or claim that may be made by its manufacturer, is not guaranteed or endorsed by the publisher.
